# Modeling organic carbon loss from a rapidly eroding freshwater coastal wetland

**DOI:** 10.1038/s41598-019-40855-5

**Published:** 2019-03-12

**Authors:** Katherine N. Braun, Ethan J. Theuerkauf, Andrew L. Masterson, B. Brandon Curry, Daniel E. Horton

**Affiliations:** 1 Illinois State Geological Survey, Prairie Research Institute, University of Illinois at Urbana-Champaign, 615 E Peabody Drive, Champaign, IL 61820 USA; 2 Program in Environmental Sciences, Northwestern University, 2145 Sheridan Road, Evanston, IL 60208 USA; 3 Department of Earth and Planetary Sciences, Northwestern University, 2145 Sheridan Road, Evanston, IL 60208 USA; 4 Department of Earth and Environmental Sciences, University of Illinois at Chicago, 845 W Taylor St, Chicago, IL 60607 USA

**Keywords:** Environmental sciences, Natural hazards

## Abstract

Shoreline erosion can transition freshwater coastal wetlands from carbon sinks to carbon sources. No studies have explored the impacts of coastal geomorphic processes on freshwater wetland carbon budgets. To do so, we modified a saltmarsh carbon budget model for application in freshwater coastal wetlands. We validated the model with data from a shoreline wetland in the Laurentian Great Lakes. The model generates the carbon budget by differencing carbon export and carbon storage. The inputs for carbon storage are the carbon inventory and maximum wetland age. Inputs for carbon export include erosion rates and overwash extent. The model demonstrates that the wetland examined in this study transitioned to a source of carbon during periods of erosion. In fact, the net carbon export between 2015 and 2018 was 8.1% of the wetland’s original carbon stock. This study indicates that geomorphic change can dictate whether and how freshwater coastal wetlands serve as sources or sinks for terrestrial carbon, and that carbon stocks can fluctuate on a geologically rapid timescale. We recommend that such geomorphic processes be considered when developing carbon budgets for these marginal environments. Furthermore, the carbon budget model refined in this study can be used to prioritize wetlands in land management and conservation efforts.

## Introduction

Freshwater wetlands are a key component of the global carbon cycle as they account for 90 to 95% of the world’s wetlands and have some of the highest rates of wetland soil organic carbon sequestration^1^. In the United States, freshwater wetlands are estimated to store $9.6\ast {10}^{15}$ g C, which is nearly ten times the quantity of carbon stored in tidal saltwater sites^2^. Coastal freshwater wetlands store carbon in both aboveground and belowground biomass. The sustainability of these carbon sinks, however, is threatened by landscape changes such as erosion, land conversion, and urbanization. Furthermore, the role of geomorphic changes, such as erosion and overwash, in the freshwater wetland carbon cycle is poorly understood. These geomorphic changes are critical for assessing the value and vulnerability of these landscapes because they can alter the area of the wetland as well as export previously stored carbon^3,4^.

The sustainability of freshwater wetland ecosystem services, such as carbon storage, flood control, and water quality regulation, relies on conserving or increasing wetland area. Shoreline erosion and wetland burial by overwash can reduce the carbon stock, which is the total amount of organic carbon stored within the wetland soil, in coastal wetlands by exporting organic carbon-rich sediment from wetlands and reducing wetland area^3–5^. Shoreline erosion exports carbon from the wetland stock and overwash deposition narrows the wetland by burying it with sand, which reduces the capacity of a wetland to actively sequester carbon^4,6^. The influence of these geomorphic processes on wetland carbon must be considered in order to fully evaluate coastal wetland carbon budgets. In this manuscript we build on previous studies that have explored the impacts of geomorphic processes on the carbon budgets of saltmarshes and transgressive barrier islands^3,4,7,8^. Our work focuses on the role of geomorphic change on freshwater coastal wetland carbon budgets, which has not previously been studied.

Freshwater wetlands play a vital role in the global carbon cycle as they slowly accumulate large stocks of carbon^9,10^. The abundant plant biomass and anaerobic conditions in wetland soils facilitate the accumulation of large stocks of soil organic carbon^11,12^. Globally, all wetland types (i.e., tidal marshes, mangrove swamps, freshwater marshes, peatlands, etc.) contain between 455 to 700 Pg C in the upper 100 cm of soil, which accounts for 20–30% of total soil organic carbon stocks on Earth^1^. Wetlands in the United States contain 11.5 Pg C in the upper 120 cm of soil, with freshwater wetlands comprising 84% of that soil organic carbon stock^2^. Previous studies have examined and modeled upland freshwater wetlands and found a wide range of carbon accumulation rates, from low rates in peatlands (~19 g C m^−2^ yr^−1^)^13^ to higher rates in temperate, forested wetlands (~473 g C m^−2^ yr^−1^)^11^. This range of organic carbon accumulation rates, which appear to be primarily influenced by geographic location, climate, oxidizing conditions, and wetland type and vegetation, necessitates site-specific evaluations of wetland carbon storage for more accurate evaluations of global budgets.

Even with high variability in soil organic carbon accumulation rates from site to site, freshwater wetlands can function as reservoirs of carbon as long as carbon storage exceeds carbon export. In this study, we quantified soil organic carbon storage and export and define carbon export as erosion of wetland soil. Carbon is stored in wetlands through two main inputs: root and shoot humification after plant death, and root organic substances released during plant growth^14^. Changes in carbon inputs will influence the carbon accumulation rate of a wetland and therefore the total amount of carbon stored within the wetland. Examining changes in primary productivity and associated changes to carbon accumulation rates are not the focus of this study and therefore are not included in the model. Wetlands that experience changes in their carbon inputs that are not well represented by the time-averaged carbon accumulation rate we employed in our model (see Methods) will have carbon budgets that are not accurately calculated by this model.

Gaseous fluxes of methane and carbon dioxide out of wetland soils can also contribute to a wetland functioning as a carbon source^10,15,16^. Values for methane and carbon dioxide fluxes vary widely based on the geographic location and type of wetland, with values ranging from 7–333 g C m^−2^ yr^−1^ for methane fluxes^15^ and 1.5–60 g C m^−2^ yr^−1^ for carbon dioxide fluxes^16^, however, quantifying those fluxes at our study site is beyond the scope of this study. Over the course of hours to days, shoreline erosion can export wetland soil organic carbon that took centuries to millennia to accumulate^4^. Once this carbon is liberated from the wetland soil it can be remineralized and returned to the atmosphere or be reworked and redeposited in another sedimentary carbon pool^17^; however, eroded soil will lose some of its carbon during the transport process through various biogeochemical pathways. Since the goal of this study is to develop and test a freshwater coastal wetland carbon budget model, we are not evaluating the ultimate fate of the carbon after it is exported from the wetland soil. This exported carbon can no longer be considered a part of this site’s wetland carbon stock and therefore represents a reduction in this ecosystem service.

In addition to soil organic carbon storage, the Great Lakes coastal wetlands (GLCW) provide numerous ecosystem services such as providing flood control, improving water quality, providing public amenities, and functioning as habitat for various plant and animal species^18,19^. These services are threatened by habitat degradation, coastal development, and geomorphic processes, such as erosion^20,21^. Currently, around 1,730 km^2^ of coastal wetlands exist in the Great Lakes region^22^, which is only one-third of the area that existed prior to European settlement^23^. Coastal geomorphic processes, such as erosion and overwash, contribute to coastal wetland loss, but the impact of these processes on freshwater wetlands is poorly understood. This adds to the challenges land managers face in evaluating where coastal conservation and protection resources should be focused.

Shoreline erosion and overwash occur along the lakeward boundary of GLCW in response to storms and fluctuating lake levels^24–26^. As GLCW are hydraulically connected to the Great Lakes, the lakeward boundary is continuously moving in response to seasonal and annual fluctuations in lake level^19,27^. These water level fluctuations result in discontinuous shoreline retreat. High lake levels induce coastal transgression, which can result in carbon export via shoreline erosion as well as wetland burial by overwash. Erosion has been shown to facilitate fluxes of carbon out of other types of wetlands, turning saltmarshes into sources of carbon^3^ and transporting carbon away from peatlands^16^. Overwash can bury wetland plants, inhibiting carbon accumulation as the burial of plants shuts off photosynthetic carbon fixation across large swaths of productive wetlands^15^. Alternatively, low lake levels can promote the growth of beach ridges that function as barriers between wetlands and the lake, thus facilitating preservation of carbon in these wetlands^28^. At the time of this study, Lake Michigan was experiencing a period of near-record high lake levels. The average lake level in 2017 was 176.77 m IGLD 85, which is 35 cm above the long-term average of 176.42 m IGLD 85^29^. This high lake level corresponds with the historic ~30 year lake level periodicity^30^, though the future of lake level cycles due to climatic forcing is uncertain^31^.

Wetlands are dynamic ecosystems that vary across regions, thus site-specific studies are needed to quantify carbon stocks and explore how the carbon budget changes through time and space. In order to evaluate whether a given coastal wetland is a carbon sink or source, erosion and overwash must be included in the carbon budget. These geomorphic changes can narrow a coastal wetland towards a critical width where it transitions from a carbon sink to a source^3^. Simple carbon budget models are powerful tools for evaluating wetland carbon budgets with respect to coastal geomorphic processes. To date, these models only exist for estuarine-fringing saltmarshes and transgressive barrier islands^3,4^.

In this study, we modified fringing saltmarsh and transgressive barrier island carbon budget transect models for use in freshwater coastal wetlands. The primary difference between transect models of freshwater coastal wetland carbon budgets and those of saltmarshes is the impact of fluctuating freshwater lake levels. Fluctuating lake levels result in interannual and seasonal variations in the rates of erosion and overwash, with high rates during periods of high lake levels and either low or absent rates during low lake levels^28,30,32^. We captured this dynamic in our model by using a variable, sub-annual time-step. Additionally, fluctuating lake levels generate ridge and swale topography at some Great Lakes coastal sites^24^, which is a different morphology than has previously been explored using carbon budget models and must therefore be parameterized differently.

**Figure 1:**
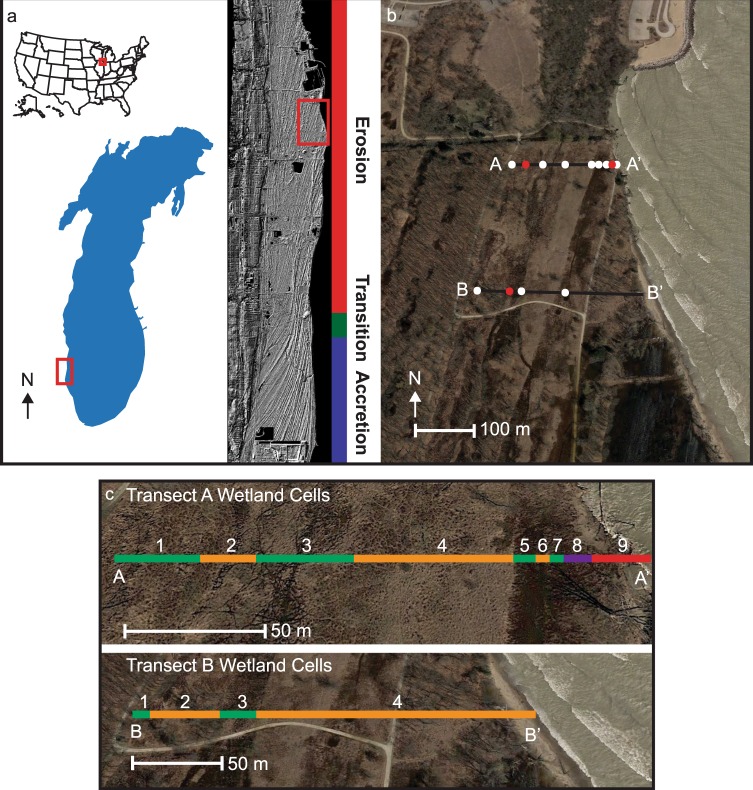
Study area map of Illinois Beach State Park (IBSP), Illinois, USA. (**a**) IBSP is a coastal state park on the western shore of Lake Michigan. The park is situated in the Zion Beach Ridge Plain, composed of curvilinear ridges and swales, as visible on the Digital Elevation Model on the right. (**b**) On the aerial photograph, white dots represent carbon core locations; red dots are radiocarbon cores. (**c**) The cell divisions for Transect A and Transect B. Green cells are wetland, orange cells are sand plain, purple cells are covered wetland, and red indicates the area of active erosion. Vector images of Lake Michigan and the United States were downloaded from Wikimedia commons (https://commons.wikimedia.org/wiki/File:Great_Lakes_Lake_Michigan.png; https://commons.wikimedia.org/wiki/File:Blank_map_of_the_United_States.PNG) and edited in Adobe Illustrator Creative Cloud. Aerial photographs used in Panels A and C were acquired from the Lake County, IL GIS Division data site https://www.lakecountyil.2180/GIS-Data. The data used to generate the Digital Elevation Model in Panel a were downloaded from the National Oceanic and Atmospheric Administration’s Data Access Viewer https://coast.noaa.gov/dataviewer/#/. Golden Software’s Surfer 13 and Adobe Illustrator Creative Cloud were used to generate Panel a. Panels b and c were generated in ArcMap 10.5 and Adobe Illustrator Creative Cloud; however, no ESRI data were used to make this figure.

The model computes changes in carbon storage and the carbon stock in response to variations in shoreline retreat rates and overwash dynamics. We test the model with field data collected from a beach ridge plain on the southwestern Lake Michigan shoreline that experienced erosion and overwash during a period of sustained high lake level (Fig. 1). The past, present, and future carbon storage potential of the site is examined in relation to recent and historic erosion and overwash events. This carbon budget model provides coastal managers with a simple means of estimating the carbon stock of wetland sites and can be used to aid in prioritization of restoration and conservation efforts.

## Methods

### Model parameters

**Figure 2:**
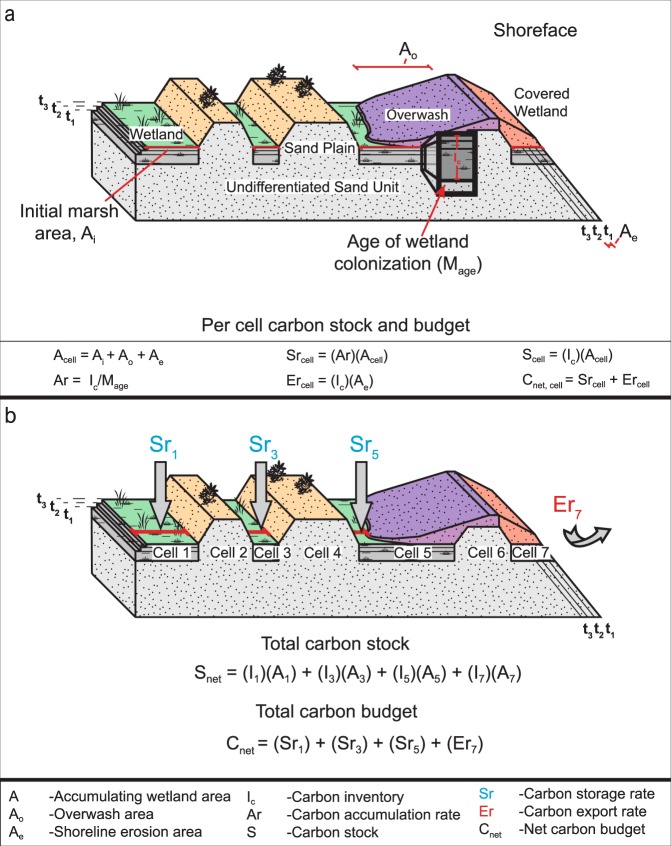
(**a**) Conceptual model of the freshwater coastal wetland carbon budget model (not to scale). (**b**) Depiction of the cell concept and summation of carbon stock and carbon budget across wetland extent.

The freshwater coastal wetland carbon budget model (Fig. 2) was developed by adding the geomorphic change parameters from a transgressive barrier island carbon budget transect model^4^ to elements of a fringing saltmarsh carbon budget model^3^. The model is composed of interacting cells along a 1 m wide shore-normal transect that account for geomorphic heterogeneity across a study site. Carbon export in the model is only occurring at the aquatic boundary of the site; carbon storage in the freshwater wetland model is a function of the carbon accumulation rate and wetland area, which changes in response to shoreline erosion and overwash. The freshwater coastal wetland carbon budget, *C*_*net*_, is the difference between the amount of carbon exported through erosion, *Er*, and the amount of carbon stored across the active wetland platform, *Sr* (Equation 1; see Table 1 for units on all variables).

1 $$\begin{array}{c}{C}_{net}=Sr+Er\end{array}$$

**Table 1:** Variables, descriptions, and units for model.

Variable	Description	Units
C_net_	Net carbon budget	g C
Sr	Carbon storage rate	g C yr^−1^
Er	Carbon export rate	g C yr^−1^
Ar	Carbon accumulation rate	g C m^−2^ yr^−1^
A	Wetland area	m^2^
I_cell_	Carbon inventory	g C m^−2^
M_age_	Wetland age	years
Δ*t*	Time step	years

The carbon budget is calculated separately for each discrete cell across the transect and then the budget values for each cell are summed (Fig. 2). Cells are defined by the geomorphology of the study site. We identified three geomorphic cell types at our study site: actively productive wetland, overwash buried wetland, and lacustrine sand plain. Carbon accumulation only occurs in the active wetland. Buried wetland cells contain a carbon-rich organic unit of wetland sediment that is covered by a layer of sand or gravel (i.e., overwash deposit), thus it cannot accumulate additional carbon. The sand plain cells by definition do not contain any wetland material, thus they do not have a carbon stock and neither store nor export carbon.

The total carbon budget and total carbon stock are calculated for each time-step by computing the budget and stock for each cell and then summing the individual cell data across the entire cross-sectional transect. A positive carbon budget signifies net storage of carbon across the wetland and indicates that the wetland is a carbon sink; a negative carbon budget denotes a net export of carbon from the wetland and that it is a carbon source. The carbon stock is a function of the carbon budget. If the carbon budget is negative, then the carbon stock will decrease, but if the carbon budget is positive, then the carbon stock will increase^4^.

The carbon accumulation rate, *Ar*, and the area of the active wetland, *A*_*n*_, are used to quantify the carbon storage term:

2 $$\begin{array}{c}S{r}_{cell}=(Ar)({A}_{n})\end{array}$$

The carbon accumulation rate is the carbon inventory of that cell, *I*_*cell*_, divided by the age of the wetland, *M*_*age*_^33^:

3 $$\begin{array}{c}Ar=\frac{{I}_{cell}}{{M}_{age}}\end{array}$$

The carbon inventory is derived from elemental analysis of wetland sediment and the wetland age is derived from radiocarbon dating of basal wetland material; the carbon inventory is the total amount of carbon per unit area^33^. The organic carbon accumulation rate is assumed constant through time; however, carbon accumulation has likely varied throughout the history of the wetland on interannual and seasonal cycles in response to water level variability, vegetation dynamics, and climate change^34,35^. We assume this variability is encompassed in the carbon accumulation rates since the carbon inventory and wetland age are generated individually for each model cell (see Supplemental Information).

The area of the wetland is the sum of the wetland area from the previous time-step, *A*_*n-1*_, and any changes due to overwash, *A*_*o*_, or shoreline change, *A*_*e*_:

4 $$\begin{array}{c}{A}_{n}={A}_{n-1}+{A}_{o}+{A}_{e}\end{array}$$

The initial wetland area as well as the extent of overwash and shoreline change can be measured from historical aerial photographs and/or high-resolution topographic surveys, such as global positioning system (GPS), Light Detection and Ranging (LIDAR), and structure-from-motion (SfM) surveys using small-unoccupied aerial systems. As our study site has limited alongshore variability in wetland vegetation, morphology and erosion rates and the primary intent of our study is to model temporal variability in the carbon budget, we used an alongshore length of 1 m to obtain our wetland areas. At sites with greater alongshore heterogeneity, cell areas will need to be individually measured per time-step to accurately model the influence of variably shaped overwash and erosion.

Carbon export is determined by multiplying the carbon inventory by the shoreline change rate:

5 $$\begin{array}{c}Er={I}_{cell}\ast \frac{{A}_{e}}{\Delta t}\end{array}$$

The shoreline change rate is the extent of the shoreline change divided by the time-step. In our model, eroded carbon is considered to be completely removed from the wetland system and is no longer part of that site’s carbon stock; however, the ultimate fate of that carbon is unclear. Eroded carbon in freshwater lakes can be remineralized, sequestered in lake sediments as organic carbon, or emitted to the atmosphere as carbon dioxide or methane^17^.

We estimate carbon storage, *Sr*, and carbon export, *Er*, as annual rates. The total carbon budget of the wetland can be expressed simply as the sum of carbon storage and carbon export if the time-step of the model is in yearly increments. In this model application, we used geomorphic change data from time-steps that were variable and generally shorter than one year. These time steps ranged from seven days to twelve months and were chosen to capture carbon dynamics associated with erosion and overwash events. When the time-step of the model is variable or not in yearly increments, the carbon budget must be scaled by the time-step:

6 $$\begin{array}{c}{C}_{net}=(Sr+Er)\ast \Delta t\end{array}$$

This modification allows the model to better characterize the effects of event-driven processes, such as overwash and erosion events associated with storms or seasonal water level changes.

### Study site

We tested the freshwater coastal wetland carbon budget model at Illinois Beach State Park (IBSP) in northeastern Illinois, USA, using field and laboratory analyses to gather the required parameters. IBSP is part of the 19 km long Zion Beach Ridge Plain, which is located along the southwestern shore of Lake Michigan and is composed of beach ridges, dunes, sand plains, and inter-ridge, or swale, wetlands (Fig. 1). The protective barriers of the beach ridges allow for the accumulation of thick organic material in swale wetlands^36^. These wetlands contain ecosystems that provide valuable services, such as storm water retention, nutrient filtration, animal habitat, and carbon storage^37^.

**Figure 3:**
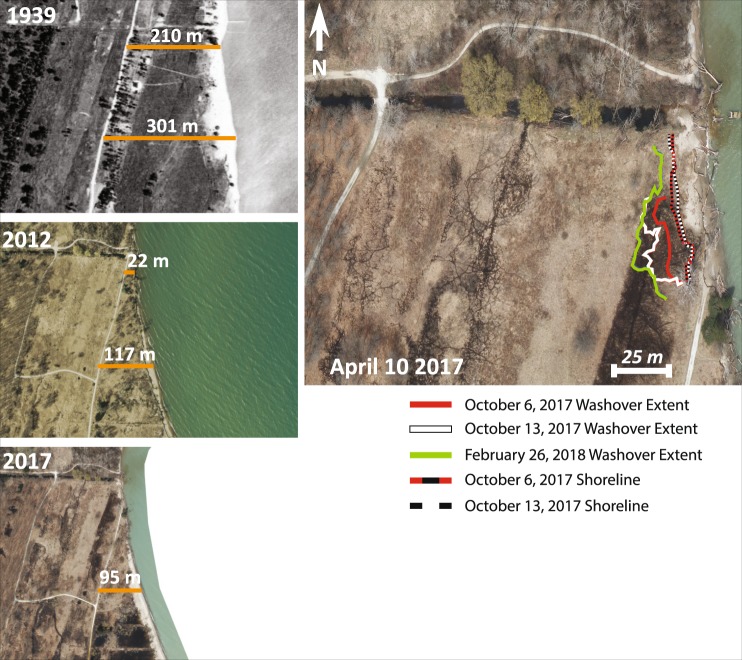
(Left) Historic aerial photographs displaying the long-term erosional trend of the North Unit of Illinois Beach State Park. Orange lines depict change in the width of the beach ridge fronting wetlands at Transects A and B since 1939. (Right) GPS derived washover and shoreline change for the shoreface of Transect A. Aerial photographs used in this image were acquired from the Lake County, IL GIS Division data site https://www.lakecountyil.gov/2180/GIS-Data and ArcMap 10.5 and Adobe Illustrator Creative Cloud were used to generate the figure; however, no ESRI data were used to make this figure.

Our study site, which is located in the northern section of IBSP, has a documented history of rapid erosion and shoreline recession throughout the 20^th^ century (Fig. 3)^38^. Annual erosion rates on average are 3 m/yr and rates as high as 60 m/yr have been documented^39^. Particularly high rates of erosion, such as those seen during the 2015–2017 study period, correspond with periods of high lake level, which varies seasonally and on interannual, multi-decadal, and centennial scales^19,40^.

We sampled and modeled carbon budgets along two shore-normal transects with a 1 m alongshore length (A and B). These two transects sample the eroding beach as well as the ridge and swale topography. Transect A was selected because previous site visits revealed a wetland unit eroding on the foreshore during high lake levels in the summer of 2017. Transect B is ~230 m south of Transect A and the wetland at this site is still protected on its lakeward boundary by a ~200 m-wide beach ridge; however, rapid shore retreat is eroding away the ridge. This is similar to the geomorphic history of Transect A over the last few years, thus Transect A’s current carbon budget dynamics represent the future of Transect B if erosion of the beach ridge continues (Fig. 3).

### Field sampling

**Figure 4:**
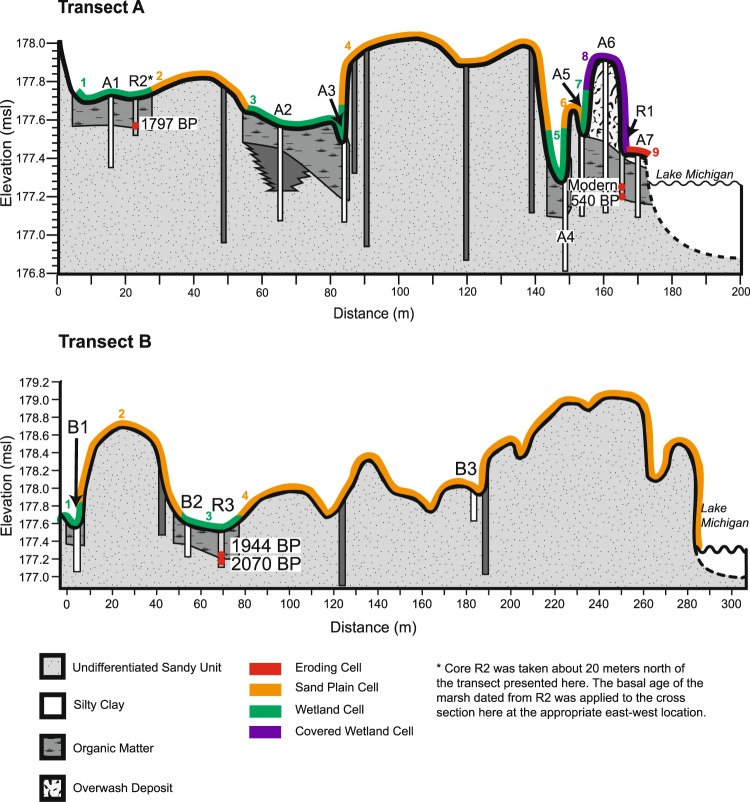
Stratigraphic cross-section of Transect A (top) and Transect B (bottom), based on topography from October 2017. Cores in white were used to parameterize the carbon budget and cores in light gray were used for radiocarbon dating. Dark grey cores denote augering locations. Transect and core locations can be found in Fig. 1.

Sediment cores were collected along the two transects in fall 2017 for carbon analysis and radiocarbon dating. All cores were collected by hammering PVC core barrels into the wetland to the point of refusal. Additional augering was conducted through the sand plains to further define the sedimentology and stratigraphy of the study site (Fig. 4). The location and elevation of coring sites were gathered with a Trimble GEO7X Real-time Kinematic Global Positioning System (RTK-GPS) with ~2 cm horizontal and vertical accuracy. A topographic profile was collected with the RTK-GPS across the transects to map the elevation of the transects between cores. Overwash extent, which is the most landward position of the overwash deposit, and the wetland shoreline position, defined as the lakeward extent of outcropping wetland material, were mapped using the RTK-GPS. Shoreline and overwash measurements with the RTK-GPS were collected on April 10, 2017; October 6, 2017; October 13, 2017; and February 26, 2018.

### Laboratory methods

#### Carbon Content

Carbon content of the sediment samples was measured on a Costech Instruments Elemental Combustion System, paired with Delta V Plus Isotope Ratio Mass Spectrometer and a Conflo IV in the Integrated Laboratories for Earth and Planetary Science at Northwestern University. Samples were decarbonated to remove inorganic carbonates by acidification with hydrochloric acid. The acidified samples were dried in an oven at 70 °C for at least two days and then were reweighed to determine the mass loss associated with the acidification of CaCO_3_. The dried samples were then weighed into tin capsules for analysis in the Elemental Combustion System. Samples that visually appeared organic rich were weighed out to 1.6 mg; sandy samples were weighed to 35 mg. Duplicates of 20% of the samples were run for error associated with both acidification and the Elemental Combustion System. Carbon content error associated with acidification is ±1.61% and error associated with the Elemental Combustion System is ±2.25%. Total carbon content error is ±2.00%.

#### Radiocarbon Dating

Radiocarbon cores were split and sectioned into 2 cm sediment samples in the same manner as the carbon cores. The deepest sample containing organic material, which was assumed to be the oldest wetland material, was disaggregated in boiling water and washed through a fine-meshed two-sieve stack. The washed residue was examined under a dissecting microscope; plant macrofossils, such as seeds and leaves of emergent and terrestrial plants and wood fragments, were selected for radiocarbon analysis. Submergent aquatic plant macrofossils (i.e., *Naja* (pondweed) seeds) were avoided as these plants may derive their carbon from dissolved carbon from Paleozoic carbonate rock (i.e., the “hard water effect”)^41^. Fossils were stored in 18% HCl, and submitted for radiocarbon dating at the University of California Irvine W.M. Keck Carbon Cycle Accelerator Mass Spectrometer facility. The basal wetland ages were calibrated using Calib 7.1 and IntCal 13^42^ and are assumed to represent the onset age of wetland colonization^43^.

#### Geomorphic Change

ArcGIS was used to map shoreline changes and overwash at each site (Fig. 3). Geo-rectified historic aerial photographs from the Lake County, Illinois Planning, Building, and Development Department were analyzed in ArcGIS, providing a yearly record of geomorphic change from 2015–2017. The RTK-GPS field data collected in 2017–2018 provide a more temporally detailed quantification of geomorphic change. Shoreline change and overwash extent were measured along two shore-normal transects on either side of the coring Transect A and provide an average rate of change as well as a maxima and minima for erosion and overwash around the studied transect. The shoreline change parameter was determined from these data as the average change in the shoreward extent of the wetland along the transect since the previous time-step. The overwash extent was defined as the average change in the landward extent of the overwash deposit since the previous time-step.

## Results

### Carbon content, inventory, and wetland age

The wetland unit sampled at Transect A was ~20–30 cm thick and contained organic carbon values ranging from 1–34% C depending on depth (average: 14.2% C). The landward wetlands at Transect A have a basal wetland age of 1,797 cal yrs BP, and the shoreface basal wetland age is 540 cal yrs BP. Percent organic carbon decreases to 1–5% at the base of the wetland unit in all cores except A3, which had a carbon content of 14% C at the base of the wetland unit where a sharp contact was observed between the overlying wetland unit and the basal sand unit.

Transect B contains a swale wetland of 20–26 cm thickness and is similar to the landward portion of Transect A in its carbon content and age (2–35% C; 2,070 cal yrs BP). The carbon content data clearly differentiate the sand plain sediment, core B3, from the wetland sediment in all other cores. The average soil organic carbon content of B3 is 5% C, well below the average of the two wetland cores in Transect B, 28.5%. All Transect A cores have values aligning with the wetland B1 and B2 cores, indicating that all cores besides B3 sampled the wetland unit.

The carbon inventory of each wetland cell was determined by summing the organic carbon content of the carbon core contained within that cell. In general, the more landward cores had lower carbon inventories than the lakeward cores, with the exception of A3, which is likely due to that core having the thickest wetland unit of all the cores. Carbon inventories for both transects are listed by core in Table 2 and range from 10 kg C m^−2^ to 24 kg C m^−2^ for Transect A and 11 kg to 20 kg C m^−2^ for Transect B. These carbon inventories fall outside the range of previously published soil organic carbon inventories for temperate wetlands (30 kg C m^−2^ to 120 kg C m^−2^), and are closer to the values reported for temperate forests (5 kg C m^−2^ to 25 kg C m^−2^)^44^. However, cores A3, A7, and B1 all have greater carbon inventories than previously published inventories, possibly due to the greater age of the wetland unit (1,797–2,016 cal years BP compared to 105 cal years BP in Pearse *et al*. 2018). The carbon inventory of all cells is the inventory of the carbon core taken within the cell. Transect A cell 3 is the only exception to this because it contains two carbon cores, so its carbon inventory is the average of those two cores, A2 and A3.

**Table 2:** Carbon inventory for cores in Transect A and Transect B.

Core	Carbon Inventory kg C m^−2^ (+/−2%)
A1: inland wetland	98
A2: inland wetland	69
A3: inland wetland	210
A4: shore wetland	102
A5: shore wetland	101
A6: shore wetland	116
A7: shore wetland	159
B1: inland wetland	160
B2: inland wetland	114
B3: sand plain	33

### Geomorphic change data

The shoreline position and overwash extent were determined at Transect A from 2015 to 2018 using a combination of aerial photography and RTK-GPS data (Table 3). Shoreline position and overwash extent from June 2, 2015; June 26, 2016; April 6–10, 2017; October 6, 2017; October 13, 2017; and February 26, 2018 were used for this analysis. June 2, 2015 was used as the starting point for geomorphic change as the beach ridge barrier between the wetland and the lake was fully removed via erosion between June 2015 and June 2016, leaving the wetland exposed to the effects of erosion and overwash. Between the periods of June 2016 to April 2017, 1.0 m^2^ of wetland shoreline erosion was measured with aerial photography and 7.0 m^2^ of shoreline erosion was measured between April 2017 and October 2017 using a combination of aerial photography and RTK-GPS survey data.

**Table 3:** Extent of overwash (right) and erosion (left) into cells of Transect A by date. NA: not applicable.

Date	Overwash (m^2^)				Erosion (m^2^)
	Cell 9	Cell 8	Cell 7	Cell 6	Cell 9
2 June 2015	NA	NA	NA	NA	NA
26 June 2016	NA	NA	NA	NA	NA
10 April 2017	NA	NA	NA	NA	−1.0
6 Oct 2017	−10.8	NA	NA	NA	−7.0
13 Oct 2017	−2.2	−5.0	−1.5	NA	NA
26 Feb 2018	NA	NA	−0.5	−2.7	NA

The overwash fan increased in landward extent along Transect A by 10.8 m^2^ between April 2017 and October 6, 2017. This increase in overwash extent likely represents deposition from multiple storm events over those periods rather than a gradual accumulation of overwash. A single storm event occurred between October 6 and October 13, 2017, which resulted in the fan extending landward another 8.7 m^2^. Between October 13, 2017 and February 26, 2018, the fan extended further landward by 3.2 m^2^, likely in response to multiple northeast wave events that occur frequently during the winter. The overwash deposit mapped on October 13, 2017 buried the last remaining wetland in Cell 9, along with the entirety of Cell 8 and the majority of Cell 7. The overwash deposit mapped on February 28, 2018 buried the remaining extent of Cell 7 and over half of Cell 6.

### Carbon budget model parameters

The parameters needed to calculate the carbon export rate and carbon storage rate were derived from the carbon inventory, radiocarbon dating, and geomorphic change data. The carbon accumulation rate in Transect A is highest in Cell 9 (39.5 g C m^−2^ yr^−1^) and drops steadily landward to 6.9 g C m^−2^ yr^−1^ in the most landward wetland cell, Cell 1 (see Table 4). Transect B has similar carbon accumulation rates to the landward wetlands of Transect A: 5.7 g C m^−2^ yr^−1^ for Cell 1 and 9.4 g C m^−2^ yr^−1^ for Cell 3. The highest carbon accumulation rates are similar to the average rate for northern peatlands (29 g C m^−2^ yr^−2^), while the carbon accumulation rates in general are much lower than those found in other temperate wetlands (230 to 320 g C m^−2^ yr^−2^) (Mitsch and Gosselink 2015). The difference between the landward and shoreface carbon accumulation rates likely arises because of the difference between the two age dates as well as the different carbon inventories along Transect A. The accumulation rate of the landward cells is lower as those cells are older compared to the lakeward cells. Despite differences in the age and carbon content of the landward and lakeward wetlands, the thicknesses of the units were comparable, which may reflect greater decomposition of wetland soil organic carbon in the older, landward wetlands, oxidation of organic carbon by wildfire in landward wetlands, or more terrigenous sediment arriving from the landward side of the wetland, perhaps from flooding or land use perturbations.

**Table 4:** Initial parameters for carbon budget model at Transect A (top) and Transect B (bottom). Includes carbon inventory, age, carbon accumulation rate, and cell width. NA: not applicable.

Transect	Cell	Core(s)	C Inventory (kg C m^−2^)	Age (before 2017)	C Accumulation Rate (g C m^−2^ yr^−1^)	Cell Area (m^2^)
A	1	A1	98	1858	53	25
	2		NA	NA	NA	20
	3	Average: A2 and A3	140	1858	75	35
	4		NA	NA	NA	57
	5	A4	102	607	168	8
	6		NA	NA	NA	4
	7	A5	101	607	166	2
	8	A6	116	607	191	5
	9	A7	160	607	264	21
B	1	B1	163	2083	78	10
	2		NA	NA	NA	40
	3	B2	114	2083	55	30
	4		NA	NA	NA	200

At Transect A, carbon export occurred in response to erosion between June 2016 and October 2017. Carbon export was highest during the summer of 2017 through October 6, 2017, when the Transect A shoreface eroded 7.7 m^2^, but then later dropped to zero when erosion ceased after October 6, 2017. Carbon storage steadily decreased through June 2016 to February 2018 as erosion removed wetland that could store carbon and as overwash covered productive wetland. While carbon export stopped after October 13, 2017, overwash continued to bury wetland, thus shutting down new carbon accumulation across those portions of the wetland.

### Net carbon budget and stock

**Figure 5:**
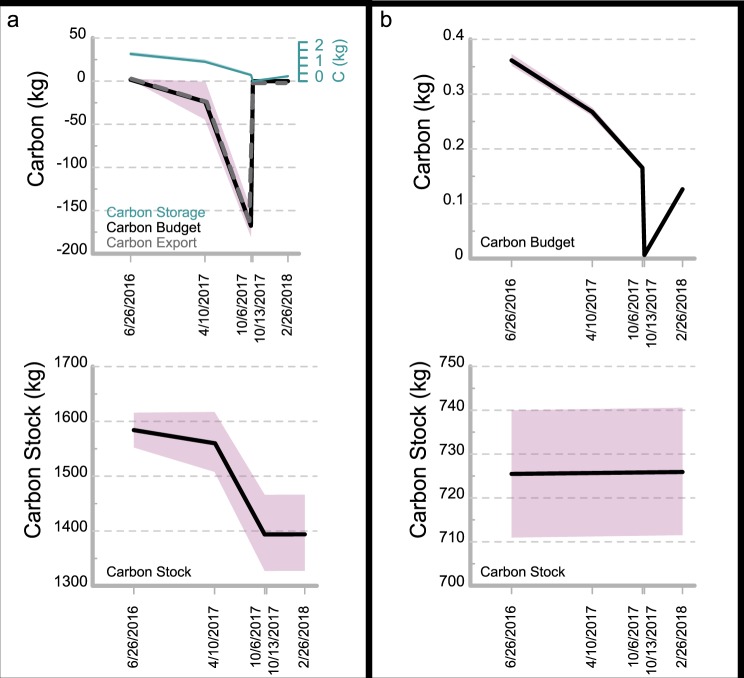
(**a**) Graphs of net carbon budget in black with carbon storage in green and carbon export in dashed grey (top), and carbon stock (bottom), at Transect A from June 2015–February 2018. Pink shaded areas indicate model error measured using high and low values for the carbon inventory, wetland age, and shoreline change. (**b**) Graphs of net carbon budget (top) and carbon stock (bottom) at Transect B from June 2015 – February 2018. No carbon export occurred at Transect B, so the carbon budget line is the same as the carbon storage line.

The carbon budget and carbon stock for both transects were calculated from 2015 to 2018 using the model parameters derived from the field and laboratory analyses (Fig. 5). The wetland at Transect A switched between functioning as a sink and source between 2015 and 2018. The carbon budget model for Transect A begins in June 2015 with a carbon stock of 1,582 kg C. From June 2, 2015 to June 26, 2016, the wetland functioned as a sink of carbon, storing 1.75 kg C. The wetland at Transect A transitioned to a carbon source between June 26, 2016 and October 6, 2017 and lost 229 kg C. From October 6, 2017 to February 26, 2018, the wetland functioned as a small carbon sink, storing only 0.24 kg C. This occurred despite large overwash events because there was no shoreline erosion during this period, thus no carbon export. During the entire duration of this study, from June 2, 2015 to February 28, 2018, this section of the wetland lost in total 188 kg C from its carbon stock, which is 12% of the original carbon stock. The stock decreased from 1,582 kg C to 1,394 kg C. If no erosion or overwash had occurred during the model run, the carbon stock would have increased by 4.5 kg C, which is an increase of 0.28%. The model at Transect A is run for a 1 m alongshore length; however, the total alongshore length of wetland that was exposed to shoreface processes during the model run was ~58 m. Therefore, the reduction in carbon stock of the entire threatened shoreface wetland at Transect A is ~10,900 kg C.

Transect B did not experience any wetland erosion between 2015 and 2018 so there is zero carbon export and the budget is completely controlled by carbon storage (Fig. 5). This site functioned as a carbon sink throughout the entire study. The carbon stock is steadily increasing given that the carbon budget is always positive for Transect B. The carbon stored between 2015 and 2018 was 0.93 kg C, a 0.13% increase from the original carbon stock of 725 kg C.

## Discussion

Across timescales ranging from days to months, geomorphic processes are removing carbon that accumulated in swale wetlands over thousands of years. Even though Transect A in our study did at times function as a carbon sink, the increase in carbon input during these periods was not enough to compensate for the carbon lost due to erosion. In total, 12% of the original carbon stock at Transect A was removed over the course of the study period, from June 2015 to February 2018. The majority of this loss of carbon (89%) occurred over six months in the summer of 2017. This rapid loss of carbon is large compared to the current carbon accumulation rates: the remaining wetland at Transect A would need to remain undisturbed for 353 years to replace the carbon lost over that six month period. There is a clear temporal imbalance between the rate at which carbon is accumulating in the wetland and the rate at which it is being eroded. In addition to the loss of carbon stock, other ecosystem services that are contingent upon wetland area, such as flood control and habitat quality, are threatened by wetland narrowing due to erosion and overwash.

In contrast to Transect A, the wetlands along Transect B are sustained sinks of carbon. The beach ridge protects these wetlands from erosion and overwash. Without erosion and overwash facilitating carbon export, the carbon stock at Transect B steadily grew through time. This highlights the importance of minimizing erosional and overwash processes if preservation of carbon stocks and other similar ecosystem services is a priority.

At IBSP, erosion of wetland material controls whether the wetland is a net sink or source of carbon. As erosion occurred along Transect A, more carbon-rich sediment was removed from the system than was stored through carbon accumulation. When overwash occurred, it did not immediately influence whether the site transitioned from a carbon sink to a source. Rather, overwash buried the wetland and reduced the wetland area available for carbon storage. Erosion and overwash reduce wetland area, which decreases the carbon storage rate and limits the potential for the wetland carbon stocks to recover after carbon export events. The rate of wetland erosion appears to be the ultimate driver of how quickly a site converts to a carbon source. If a wetland site experiences more persistent, small magnitude erosion, the carbon stock of that site will slowly be depleted as the wetland narrows. Storm events or anthropogenic disturbances superimposed upon this slow transition could accelerate the transition of a site towards a carbon source.

The impact of seasonal high lake levels was captured in the Transect A carbon budget. Between April 2017 and October 2017, 7.0 m^2^ of wetland erosion exported 11% of the original carbon stock. As lake level lowered in the fall, no further erosion was recorded. Overwash, however, continued to occur during fall storm events. The impact of a storm on October 10, 2017 is clearly seen in the expansion of the overwash fan at Transect A recorded on October 13 and the associated reduction in carbon storage (Fig. 5).

Seasonal and interannual cycles of high Great Lakes water levels will induce erosion and overwash that excavates coastal wetlands and reduces the wetland carbon storage potential. These processes may cease when water level lowers, but if the wetland area was reduced by erosion and overwash, carbon storage will be permanently reduced. Furthermore, the annual erosion rate in the North Unit of IBSP is 3 m/yr, which indicates that shoreline erosion is a chronic problem in this region. Wetlands can recolonize emergent coastlines, but the emergent wetlands are drowned and returned to open water during high lake level periods^45^. Given the short periodicity of lake level fluctuations in the Great Lakes (~30 years)^30^, it is unlikely that wetlands will be able to fully recover the carbon stock and area lost to geomorphic change during high lake levels. Eighty years of historical, aerial photography for Transect A do not suggest any large-scale wetland re-colonization following erosion during periods of high lake levels (Fig. 3).

It is possible that coastal wetlands have been eroded and recolonized cyclically in the past. The lakeward wetlands in Transect A are dated at 540 BP, which is significantly younger than the landward wetlands in that transect (1,800 BP). This difference in age could be due to a cycle of erosion and deposition that reworked the landward wetlands as lake level fell since the Algoma stage (3,800–2,500 BP)^28^. Our carbon budget model, however, is intended to represent modern processes across management-relevant time-scales (i.e., years to decades). Our study site only became vulnerable to coastal processes since 2015 when the protective lakeward beach ridge eroded. As there is no evidence in aerial photographs from the past century that wetlands recolonized areas that were eroded and overwashed during past high lake level cycles, we assume they will not recolonize at this study site in the coming decades. No recovery is likely for coastal freshwater wetland carbon lost due to coastal geomorphic processes across management-relevant time-scales and thus should be considered permanently lost for the purposes of conservation and management.

The two transects examined at IBSP depict different wetland morphologies that are common throughout the Laurentian Great Lakes and other large freshwater basins: Transect A contains open coast wetland that is directly exposed to lacustrine shoreline processes and Transect B contains wetland that is protected from those processes by a wide beach ridge. The current carbon budget dynamics occurring at Transect A can be considered a future analog for Transect B as erosion that removed the beach ridge at Transect A is currently impacting the protective beach ridge at Transect B. Sustained shoreline erosion will likely reduce the beach ridge width, eventually transitioning the Transect B wetlands from protected wetlands to open coast wetlands and threatening the carbon stocks. In the future, the wetland at Transect A will experience periods of rapid carbon loss and reduced storage potential when wetland cells are exposed, followed by periods of carbon storage when the sand plains are exposed at the shoreface. Given the order of magnitude imbalance between carbon storage and carbon export rates, however, it is unlikely that the carbon stock will recover during periods when sand plains are exposed on the shoreface.

The sub-annual time-step utilized in this study is useful for examining event-driven carbon budget dynamics at all types of coastal wetlands, including saltmarshes. The saltmarsh carbon budget models that this model was based on used annual rates of overwash and erosion^3,4^. Annual rates normalize any smaller-scale carbon budget dynamics occurring during the year, which makes it difficult to discern the specific events that result in carbon export. Therefore, we refined the model to use the magnitude of erosion and overwash that occurs during a discrete time-step to parameterize carbon storage, rather than average rates of erosion and overwash. This finer temporal scale model more accurately captures sub-annual and event-scale changes in the carbon budget, which allows users to identify the specific events that transition a wetland to a sink or source

Geomorphic impacts on freshwater coastal wetland carbon budgets have not been previously considered, yet the results of our study indicate that these impacts can negatively influence the carbon budget of these landscapes. While morphologies differ from site-to-site, all coastal wetland sites are influenced by geomorphic processes to some degree. Given the prevalence of other beach ridge complexes and coastal wetlands both within the Great Lakes^22,30,46–48^, as well as in other large freshwater systems globally, it is likely that the high rates of wetland retreat and carbon loss at this study site are not anomalous. Local and regional wetland carbon budgets should include the influence of geomorphic processes in order to accurately capture both the present and future carbon dynamics. This study focuses on the geomorphic processes of erosion and overwash at a beach ridge plain; however, geomorphic influences on wetland carbon budgets also include fluvial processes at river mouths or aeolian processes near dunes. Future research aimed at discerning the ultimate fate of eroded wetland carbon would help to resolve how important coastal geomorphic processes are for wetland carbon export to the atmosphere.

Our model provides a simple method for quantifying the ecosystem service of carbon storage in freshwater coastal wetlands. Coastal land managers must decide where to devote limited resources, and the value of ecosystem services can serve as a way to prioritize different habitat types^49,50^. The carbon stock of an ecosystem and the vulnerability of that stock can be used to value wetland habitat. The knowledge of whether a wetland is susceptible to becoming a net source of carbon can aid managers in deciding which wetlands should be prioritized for conservation. This model can be adapted for use in any coastal wetland and only requires a simple set of parameters that can be generated either from existing literature or from field data. Site-specific dynamics, such as topographic complexity or spatial variability in the carbon inventory, can be easily evaluated with our model by changing the dimensions, arrangement, and/or input data for the cells.

## Conclusion

Understanding the carbon budget dynamics of freshwater coastal wetlands is necessary for making informed land management decisions to protect fragile habitats and conserve stored carbon in the face of climate change. Here, we developed a freshwater coastal wetland carbon budget model with three cell types to account for landscape heterogeneity. The model was evaluated at a beach ridge complex along southwestern Lake Michigan in the Laurentian Great Lakes, which experiences erosion and overwash during cyclical periods of high water level. Erosion of shoreface wetland material led to rapid loss of carbon and transitioned the wetland from a sink to a source of carbon. 8% of the combined carbon stock of the wetlands in Transect A and B was lost between 2015 and 2018, with most of this loss occurring during a six-month period in 2017. Export of carbon occurs during erosional events, yet even when carbon export ceases, the original carbon stock cannot be recovered as the export of carbon is two orders of magnitude greater than carbon storage. This model provides a simple tool for quantifying the carbon stocks of a wetland, which can be used for conservation and restoration efforts.

## Supplementary information

Supplementary Information

## Data Availability

The datasets generated and analyzed during the current study are available from the corresponding author on reasonable request.
