# Author Correction: Modeling organic carbon loss from a rapidly eroding freshwater coastal wetland

**DOI:** 10.1038/s41598-020-60881-y

**Published:** 2020-03-06

**Authors:** Katherine N. Braun, Ethan J. Theuerkauf, Andrew L. Masterson, B. Brandon Curry, Daniel E. Horton

**Affiliations:** 1 Illinois State Geological Survey, Prairie Research Institute, University of Illinois at Urbana-Champaign, 615 E Peabody Drive, Champaign, IL 61820 USA; 2 Program in Environmental Sciences, Northwestern University, 2145 Sheridan Road, Evanston, IL 60208 USA; 3 Department of Earth and Planetary Sciences, Northwestern University, 2145 Sheridan Road, Evanston, IL 60208 USA; 4 Department of Earth and Environmental Sciences, University of Illinois at Chicago, 845 W Taylor St, Chicago, IL 60607 USA

Correction to: *Scientific Reports* 10.1038/s41598-019-40855-5, published online 12 March 2019

The original version of this Article contained errors.

The authors identified the following errors in the published version of the Article: radiocarbon dates were incorrectly reported, and the carbon inventory data was incorrectly processed. The authors repeated processing of the carbon inventory data, and updated the carbon accumulation rate and carbon budget model. While the correction affects the scale of patterns observed, the pattern itself remains unchanged and as such the original conclusions hold.

As a result of these corrections, in the Abstract,

“In fact, the net carbon export between 2015 and 2018 was 10% of the wetland’s original carbon stock.”

now reads:

“In fact, the net carbon export between 2015 and 2018 was 8.1% of the wetland’s original carbon stock.”

In the Results, section ‘Carbon Content, Inventory, and Wetland Age’,

“Transect B contains a swale wetland of 20–26 cm thickness and is similar to the landward portion of Transect A in its carbon content and age (2–35% C; 2,016 cal yrs BP).”

now reads:

“Transect B contains a swale wetland of 20–26 cm thickness and is similar to the landward portion of Transect A in its carbon content and age (2–35% C; 2,070 cal yrs BP).”

and in the same section,

“Carbon inventories for both transects are listed by core in Table 2 and range from 69 kg C m^−2^ to 210 kg C m^−2^ for Transect A and 33 kg to 160 kg C m^−2^ for Transect B. Most of these carbon inventories fall within the range of previously published soil organic carbon inventories for temperate wetlands (30 kg C m^−2^ to 120 kg C m^−2^)^44^. However, cores A3, A7, and B1 all have greater carbon inventories than previously published inventories, possibly due to the greater age of the wetland unit (1,797–2,016 cal years BP compared to 105 cal years BP in Pearse *et al*. 2018).”

now reads:

“Carbon inventories for both transects are listed by core in Table 2 and range from 10 kg C m^−2^ to 24 kg C m^−2^ for Transect A and 11 kg to 20 kg C m^−2^ for Transect B. These carbon inventories fall outside the range of previously published soil organic carbon inventories for temperate wetlands (30 kg C m^−2^ to 120 kg C m^−2^), and are closer to the values reported for temperate forests (5 kg C m^−2^ to 25 kg C m^−2^)^44^.”

In Results, section ‘Carbon Budget Model Parameters’,

“The carbon accumulation rate in Transect A is highest in Cell 9 (263 g C m^−2^ yr^−1^) and drops steadily landward to 53 g C m^−2^ yr^−1^ in the most landward wetland cell, Cell 1 (see Table 4). Transect B has similar carbon accumulation rates to the landward wetlands of Transect A: 76 g C m^−2^ yr^−1^ for Cell 1 and 55 g C m^−2^ yr^−1^ for Cell 3. The lowest carbon accumulation rates are almost double the average rate for northern peatlands (29 g C m^−2^ yr^−2^), and the highest reported carbon accumulation rate is similar to averages found for temperate wetlands (230 to 320 g C m^−2^ yr^−2^) (Mitsch and Gosselink 2015).”

now reads:

“The carbon accumulation rate in Transect A is highest in Cell 9 (39.5 g C m^−2^ yr^−1^) and drops steadily landward to 6.9 g C m^−2^ yr^−1^ in the most landward wetland cell, Cell 1 (see Table 4). Transect B has similar carbon accumulation rates to the landward wetlands of Transect A: 5.7 g C m^−2^ yr^−1^ for Cell 1 and 9.4 g C m^−2^ yr^−1^ for Cell 3. The highest carbon accumulation rates are similar to the average rate for northern peatlands (29 g C m^−2^ yr^−2^), while the carbon accumulation rates in general are much lower than those found in other temperate wetlands (230 to 320 g C m^−2^ yr^−2^) (Mitsch and Gosselink 2015).”

and in the same section,

“Carbon export was highest during the summer of 2017 through October 13, 2017, when the Transect A shoreface eroded 7.7 m^2^, but then later dropped to zero when erosion ceased after October 13, 2017.”

now reads:

“Carbon export was highest during the summer of 2017 through October 6, 2017, when the Transect A shoreface eroded 7.7 m^2^, but then later dropped to zero when erosion ceased after October 6, 2017.”

In the Results, section ‘Net Carbon Budget and Stock’,

“The carbon budget model for Transect A begins in June 2015 with a carbon stock of 12.27 Mg C. From June 2, 2015 to June 26, 2016, the wetland functioned as a sink of carbon, storing 0.01 Mg C. The wetland at Transect A transitioned to a carbon source between June 26, 2016 and October 6, 2017 and exported 1.25 Mg C. From October 6, 2017 to February 26, 2018, the wetland functioned as a small carbon sink, storing only 0.0001 Mg C. This occurred despite large overwash events because there was no shoreline erosion during this period, thus no carbon export. During the entire duration of this study, from June 2, 2015 to February 28, 2018, this section of the wetland lost in total 1.24 Mg C from its carbon stock, which is 10% of the original carbon stock. The stock decreased from 12.27 Mg C to 11.04 Mg C. If no erosion or overwash had occurred during the model run, the carbon stock would have increased by 0.028 Mg C, which is an increase of 0.22%. The model at Transect A is run for a 1 m alongshore length; however, the total alongshore length of wetland that was exposed to shoreface processes during the model run was ~58 m. Therefore, the reduction in carbon stock of the entire threatened shoreface wetland at Transect A is ~72 Mg C.”

now reads:

“The carbon budget model for Transect A begins in June 2015 with a carbon stock of 1,582 kg C. From June 2, 2015 to June 26, 2016, the wetland functioned as a sink of carbon, storing 1.75 kg C. The wetland at Transect A transitioned to a carbon source between June 26, 2016 and October 6, 2017 and lost 229 kg C. From October 6, 2017 to February 26, 2018, the wetland functioned as a small carbon sink, storing only 0.24 kg C. This occurred despite large overwash events because there was no shoreline erosion during this period, thus no carbon export. During the entire duration of this study, from June 2, 2015 to February 28, 2018, this section of the wetland lost in total 188 kg C from its carbon stock, which is 12% of the original carbon stock. The stock decreased from 1,582 kg C to 1,394 kg C. If no erosion or overwash had occurred during the model run, the carbon stock would have increased by 4.5 kg C, which is an increase of 0.28%. The model at Transect A is run for a 1 m alongshore length; however, the total alongshore length of wetland that was exposed to shoreface processes during the model run was ~58 m. Therefore, the reduction in carbon stock of the entire threatened shoreface wetland at Transect A is ~10,900 kg C.”

And in the same section,

“The carbon stored between 2015 and 2018 was 0.0067 Mg C, a 0.13% increase from the original carbon stock of 5.05 Mg C.”

now reads:

“The carbon stored between 2015 and 2018 was 0.93 kg C, a 0.13% increase from the original carbon stock of 725 kg C.”

In the Discussion,

“In total, 10% of the original carbon stock at Transect A was removed over the course of the study period, from June 2015 to February 2018. The majority of this loss of carbon (89%) occurred over six months in the summer of 2017. This rapid loss of carbon is large compared to the current carbon accumulation rates: the remaining wetland at Transect A would need to remain undisturbed for 205 years to replace the carbon lost over that six month period.”

now reads:

“In total, 12% of the original carbon stock at Transect A was removed over the course of the study period, from June 2015 to February 2018. The majority of this loss of carbon (89%) occurred over six months in the summer of 2017. This rapid loss of carbon is large compared to the current carbon accumulation rates: the remaining wetland at Transect A would need to remain undisturbed for 353 years to replace the carbon lost over that six month period.”

And in the same section:

“Between April 2017 and October 2017, 7.0 m^2^ of wetland loss exported 10% of the original carbon stock.”

now reads:

“Between April 2017 and October 2017, 7.0 m^2^ of wetland erosion exported 11% of the original carbon stock.”

Finally, in the Conclusion,

“10% of the original carbon stock was lost between 2015 and 2018, with most of this loss occurring during a six-month period in 2017.”

now reads:

“8% of the combined carbon stock of the wetlands in Transect A and B was lost between 2015 and 2018, with most of this loss occurring during a six-month period in 2017.”

In addition to these changes in the text, Figure 4 and Figure 5 were corrected in the Article. The original versions of these figures are included below as Figure 1 and Figure 2.

**Figure 1:**
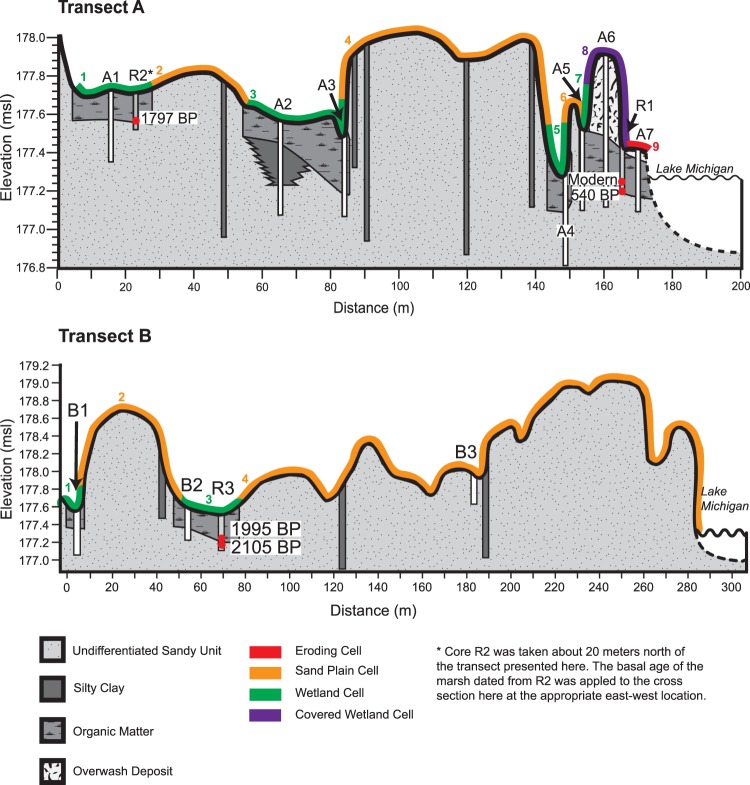
.

**Figure 2:**
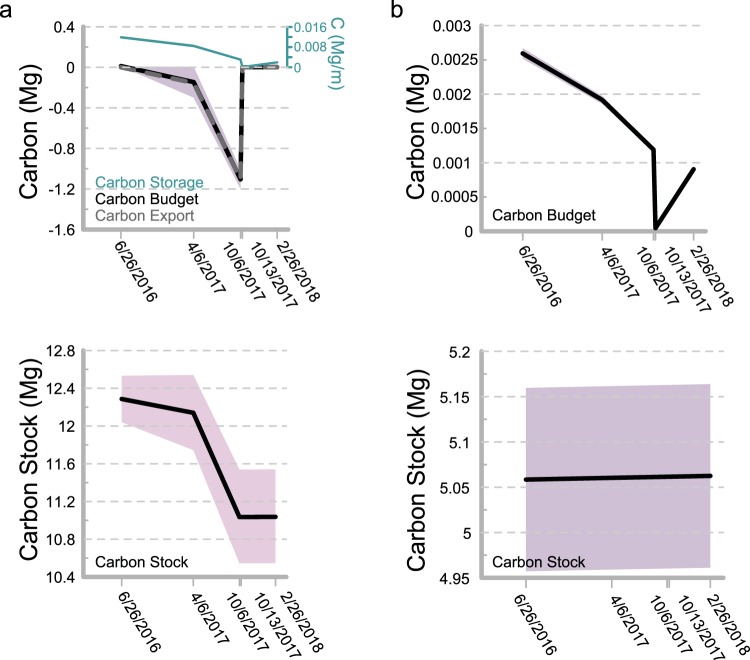
.

These errors have now been corrected in the PDF and HTML versions of the Article.

The overall conclusions of the Article are unaffected by these corrections.

