# Supplementary material for: Modeling organic carbon loss from a rapidly eroding freshwater coastal wetland
Source: Sci Rep. 2019 Mar 12;9:4204. doi: 10.1038/s41598-019-40855-5 (PMC6414515; doi:10.1038/s41598-019-40855-5)
Supplement: Supplementary file 1 — Supplementary Information [file 41598_2019_40855_MOESM1_ESM.docx]

Supplementary Information

*Scientific Reports*

**Modeling organic carbon loss from a rapidly eroding freshwater coastal wetland**

Katherine N. Braun^1,2,3^, Ethan J. Theuerkauf^1,4*^, Andrew L. Masterson^3^, B. Brandon Curry^1^ and Daniel E. Horton^3^

^1^ Illinois State Geological Survey, Prairie Research Institute, University of Illinois at Urbana-Champaign, 615 E Peabody Drive, Champaign IL, 61820

^2^ Program in Environmental Sciences, Northwestern University, 2145 Sheridan Road, Evanston IL 60208

^3^ Department of Earth and Planetary Sciences, Northwestern University, 2145 Sheridan Road, Evanston IL 60208

^4^ Department of Earth and Environmental Science, University of Illinois at Chicago, 845 W Taylor St, Chicago IL 60607

**^*^**Corresponding author: [ejtheu@illinois.edu](mailto:ejtheu@illinois.edu), 217-300-3746

Supplemental Methods

*Model Cells*

Each cell was classified based on the morphology and stratigraphy of the landscape at the time of coring and surveying. Beach ridges and sand plains are distinguished based on the scale of their morphology; beach ridges are 100s of meters wide and 1-3 m in relief, while sand plains are 10s of meters wide and <1 m in relief.

. Buried wetland cells usually occur near the shoreface, where overwash buries the nearshore, but could also occur landward in response to aeolian transportAs the carbon budget model is run, active wetland cells can transition into buried wetland cells if the wetland becomes covered. Additionally, sand plain cells can be eroded and expose landward active or buried wetland cells to shoreface processes.

*Calculation of Carbon Content*

$$\begin{aligned} wt\%TOC={wt\%TOC}_{ECS}*\left( \frac{100-wt\%TIC}{100} \right)\#\left( 1 \right) \end{aligned}$$

| $\boldsymbol{wt\%TOC}$ | True weight percent of total organic carbon (TOC) in original sample |
| --- | --- |
| $\boldsymbol{wt\%TIC}$ | Weight percent of total inorganic carbon (TIC) in sample; derived from mass loss associated with acidification of CaCO_3_ in sediment sample |
| $\boldsymbol{wt\%TOC}_{\boldsymbol{ECS}}$ | Weight percent of total organic carbon in subsample that does not include TIC; this weight percent overestimates the amount of carbon in the original sample |

Supplementary Table 1: Definitions of terms used in calculations of carbon content and error.

The sediment sample that is analyzed in the Elemental Combustion System only includes organic carbon and the inorganic carbon in the sample was dissolved in HCl prior to analysis. Therefore, the carbon content percentages derived from the Elemental Combustion System do not reflect the original composition of the sample. Supplemental equation (1) above accounts for the variation between the subsample analyzed in the Elemental Combustion System and the original sediment.

*Carbon Inventory*

The accumulation rates represent an average of the carbon dynamics of the wetland through time. The use of multiple cross-shore cells in this model alleviates some of the inaccuracies associated with assuming spatial homogeneity of carbon accumulation. While seasonality may influence carbon accumulation rates in this temperate wetland, we used a constant carbon accumulation rate to study specifically the effects of geomorphic change on wetland carbon. Given the assumptions inherent in our approach, the utility of this model should be assessed on a site-by-site basis. Freshwater wetlands with a high degree of spatial heterogeneity or a different morphology than a beach ridge plain or coastal barrier may require further model refinement.

*Calculation of Carbon Inventory*

$$\begin{aligned} Carbon inventory= \frac{wt\%TOC*sample weight}{sample volume}\#\left( 2 \right) \end{aligned}$$

The carbon inventory is the total amount of carbon in the wetland sediment, in units of kg C m^-2^.

*Impact of shoreline change on wetland carbon storage*

If the shoreline change is positive (i.e. sediment accreting at the shoreline), no carbon export is occurring, any newly deposited sediment will not contain a wetland unit and therefore is categorized as a sand plain cell. It may be possible in the future for an accreted sand plain cell to develop into a wetland cell if wetland plants colonize the sediment. On short timescales, such as those considered in this study, no appreciable carbon storage is likely to occur in any newly colonized wetland as the rates of carbon loss through erosion far outweigh rates of carbon accumulation. Thus, for the purposes of our model we consider these accreted units to function as sand plains.

*Geological History of Study Site: Illinois Beach State Park, Lake Michigan, USA*

Throughout the late Holocene, the Zion Beach Ridge Plain has been migrating southward via erosion in the northern portion of the plain, southward littoral transport of these sediments, and accretion in the southern portion of the plain^3^Construction of North Point Marina in 1989 has exacerbated erosion in the North Unit of IBSP^4^. In general, the most severe coastal erosion happens adjacent to harbor structures and other hardened points along the shoreline^5^, and this pattern is evident at our study site^4^.

Herdendorf^6^ classified wetlands throughout IBSP as coastal lagoon wetlands, which describe a wetland that is protected by a spit, ridge, or other formation produced by coastal processes. However, according to Keough et al’s^7^ Great Lakes coastal wetland classification system, the IBSP wetlands are transitioning from protected wetlands to open coast wetlands, as the protective shoreface beach ridges are eroding. Along the northernmost portion of the study site, this transition was observed between 2015 and 2016 as the lakeward-most beach ridge completely eroded in response to rising Lake Michigan water levels. This erosion directly exposed the wetland to lacustrine wave action. The model is therefore parameterized for 2015 to 2018 as the wetlands at Transect A were exposed to coastal processes during this period. Together, these two transects represent typical geomorphic changes associated with coastal transgression and will provide insight into the dynamics of carbon storage in this region as well as at other coastal landscapes, such as other beach ridge complexes or barrier islands.

*Field Methods*

Nine sediment cores for carbon analysis were collected from wetland depressions in the two transects, where the surface sediment was clearly organic. One core, B3, was collected from the sand plain to compare sand plain sediment to the wetland sediment. Three additional push-cores were used for radiocarbon analysis, one at the most landward extent of each transect and one at the shoreface of Transect A. Carbon analysis cores were 4.3 cm in diameter; radiocarbon dating cores were 10.2 cm in diameter.

*Laboratory Methods*

Prior to analysis, carbon cores were stored in a cool, dry environment. Cores were split lengthwise, photographed, and described. The organic rich units were sectioned in 2 cm bins and sampled. Wet weights of the samples were recorded, and samples were allowed to air dry in a cool, dry environment. Acetanilide and urea standards from the University of Indiana Biogeochemical Laboratories provided precision data on the Elemental Combustion System.

The total organic carbon weight percent that is measured on the Elemental Combustion System must be scaled by the amount of inorganic carbon lost during acidification to account for the mass difference and provide an accurate weight percent for the original sample. Therefore, the final error on the carbon content data must also be scaled appropriately. Variation in carbon content data is likely attributed primarily to heterogeneity within the samples.

*Model Error*

The model error was determined by running the model with the upper and lower uncertainty estimates for the carbon inventory, radiocarbon date, and geomorphic change parameters. In addition to the high and low model runs, the model was run at Transect A with no geomorphic change parameters included, to determine how the wetland carbon reservoir would have behaved without the influence of erosion or overwash. The model error is included in the plots of the carbon budget and carbon reservoir in Figure 5.

*Error Analysis of Carbon Content and Isotopic Data*

| $\begin{aligned} \boldsymbol{C=A*B\#}\left( \boldsymbol{3} \right) \end{aligned}$  $\begin{aligned} \boldsymbol{\sigma C=}\left[ \left( \boldsymbol{\sigma B} \right)^{\boldsymbol{2}}\left( \frac{\boldsymbol{\partial C}}{\boldsymbol{\partial B}} \right)^{\boldsymbol{2}}\boldsymbol{+}\left( \boldsymbol{\sigma A} \right)^{\boldsymbol{2}}\left( \frac{\boldsymbol{\partial C}}{\boldsymbol{\partial A}} \right)^{\boldsymbol{2}} \right]^{\frac{\boldsymbol{1}}{\boldsymbol{2}}}\boldsymbol{\#}\left( \boldsymbol{4} \right) \end{aligned}$  $\begin{aligned} \frac{\boldsymbol{\partial C}}{\boldsymbol{\partial B}}\boldsymbol{=A}\frac{\boldsymbol{\partial C}}{\boldsymbol{\partial A}}\boldsymbol{=B\#}\left( \boldsymbol{5} \right) \end{aligned}$  $\begin{aligned} \boldsymbol{\sigma C=}\left[ \left( \boldsymbol{\sigma B} \right)^{\boldsymbol{2}}\left( \boldsymbol{A} \right)^{\boldsymbol{2}}\boldsymbol{+}\left( \boldsymbol{\sigma A} \right)^{\boldsymbol{2}}\left( \boldsymbol{B} \right)^{\boldsymbol{2}} \right]^{\frac{\boldsymbol{1}}{\boldsymbol{2}}}\boldsymbol{\#}\left( \boldsymbol{6} \right) \end{aligned}$  $\begin{aligned} \boldsymbol{C=A}\left( \frac{\boldsymbol{100-B}}{\boldsymbol{100}} \right)\boldsymbol{=}\frac{\boldsymbol{A}}{\boldsymbol{100}}\left( \boldsymbol{100-B} \right)\boldsymbol{\#}\left( \boldsymbol{7} \right) \end{aligned}$  $\begin{aligned} \frac{\boldsymbol{\partial C}}{\boldsymbol{\partial A}}\boldsymbol{=}\frac{\boldsymbol{100-B}}{\boldsymbol{100}} \frac{\boldsymbol{\partial C}}{\boldsymbol{\partial B}}\boldsymbol{= -}\frac{\boldsymbol{A}}{\boldsymbol{100}}\boldsymbol{\#}\left( \boldsymbol{8} \right) \end{aligned}$  $\begin{aligned} \boldsymbol{\sigma C=}\left[ \left( \boldsymbol{\sigma B} \right)^{\boldsymbol{2}}\left( \boldsymbol{-}\frac{\boldsymbol{A}}{\boldsymbol{100}} \right)^{\boldsymbol{2}}\boldsymbol{+}\left( \boldsymbol{\sigma A} \right)^{\boldsymbol{2}}\left( \frac{\boldsymbol{100-B}}{\boldsymbol{100}} \right)^{\boldsymbol{2}} \right]^{\frac{\boldsymbol{1}}{\boldsymbol{2}}}\boldsymbol{\#}\left( \boldsymbol{9} \right) \end{aligned}$ |
| --- |

Supplementary equations 3-9 depict the error calculations for carbon content.

| $\begin{aligned} \boldsymbol{wt\%TOC=}\boldsymbol{wt\%TOC}_{\boldsymbol{ECS}}\boldsymbol{*}\left( \frac{\boldsymbol{100-wt\%TIC}}{\boldsymbol{100}} \right)\boldsymbol{\#}\left( \boldsymbol{10} \right) \end{aligned}$  $\begin{aligned} \boldsymbol{wt\%TOC}_{\boldsymbol{ECS}}\boldsymbol{=A wt\%TIC=B wt\%TOC=C\#}\left( \boldsymbol{11} \right) \end{aligned}$  $\begin{aligned} \boldsymbol{\sigma C=}\left[ \left( \boldsymbol{1.61} \right)^{\boldsymbol{2}}\left( \boldsymbol{-}\frac{\boldsymbol{13.86}}{\boldsymbol{100}} \right)^{\boldsymbol{2}}\boldsymbol{+}\left( \boldsymbol{2.25} \right)^{\boldsymbol{2}}\left( \frac{\boldsymbol{100-11.36}}{\boldsymbol{100}} \right)^{\boldsymbol{2}} \right]^{\frac{\boldsymbol{1}}{\boldsymbol{2}}}\boldsymbol{= \pm2.01 \%\#}\left( \boldsymbol{12} \right) \end{aligned}$ |
| --- |

Supplementary equations 10-12 depict an example of the error calculations for carbon content.

Three sets of samples were run in the Elemental Combustion System: the original samples, acidification duplicates, and ECS duplicates. The acidification duplicates are run to estimate the error associated with the acidification process. The ECS duplicates are run to estimate the error associated with the ECS system. The above error equations combine the acidification error and ECS error to produce a final error on the carbon content.

Supplemental Results

*Sedimentology and Stratigraphy*

Three distinct units were sampled in the sediment cores collected at IBSP. The deepest unit sampled in the cores was a medium-to-coarse grained, poorly sorted sand that we interpret as the sand plain defined in previous studies at IBSP^8,9^. This unit is also exposed on the surface in the low-relief ridges that comprise the North Unit of IBSP. A 10 cm thick, uniform, clay-rich unit was sampled in only one of the cores at IBSP. This unit was stratigraphically above the sand plain and was located around 110 m landward of the modern shoreline (see core A2 in Figure 4). We interpret this unit as an interdunal pond based on previous literature^11^ and a comparison to the modern landscape, where small, muddy ponds are located in deeper portions of the swales.

An organic-rich silty clay was sampled at the surface of cores collected in the swales and at depth in cores collected across the overwash fan at Transect A. This unit was interpreted as wetland based on the high-organic content, presence of wetland biomass both at the surface in swales and in the sampled soil, and similarity to previously published data^8,9^. The wetland unit extends throughout the wetland cells, as well as underneath the overwash deposit in Transect A, but not beneath the sand plains. The stratigraphic interpretations from the core and auger data were combined with topographic data from RTK-GPS to create geological cross-sections of the two transects (Figure 4).

Radiocarbon dating of basal wetland material in three locations provided ages of initial wetland colonization. The landward wetlands of Transect B are the oldest, with a basal wetland age of 2,016 cal yrs BP. The landward wetlands at Transect A have a basal wetland age of 1,797 cal yrs BP, while the shoreface basal wetland age is 540 cal yrs BP.

*Wetland Carbon Content*

The most landward cores, A1 through A5, all had peak organic carbon between 0-7 cm depth. The most landward cores (A1-A4) have greater carbon content than the lakeward cores (average 17.1% C compared to 10.5% C), presumably because they are older than the lakeward cores. Cores A6 and A7, which were collected closest to the shoreline and sampled the youngest wetland material, had peak organic carbon in the lower segments of the cores, at 15-25 cm depth.

Supplemental Figures


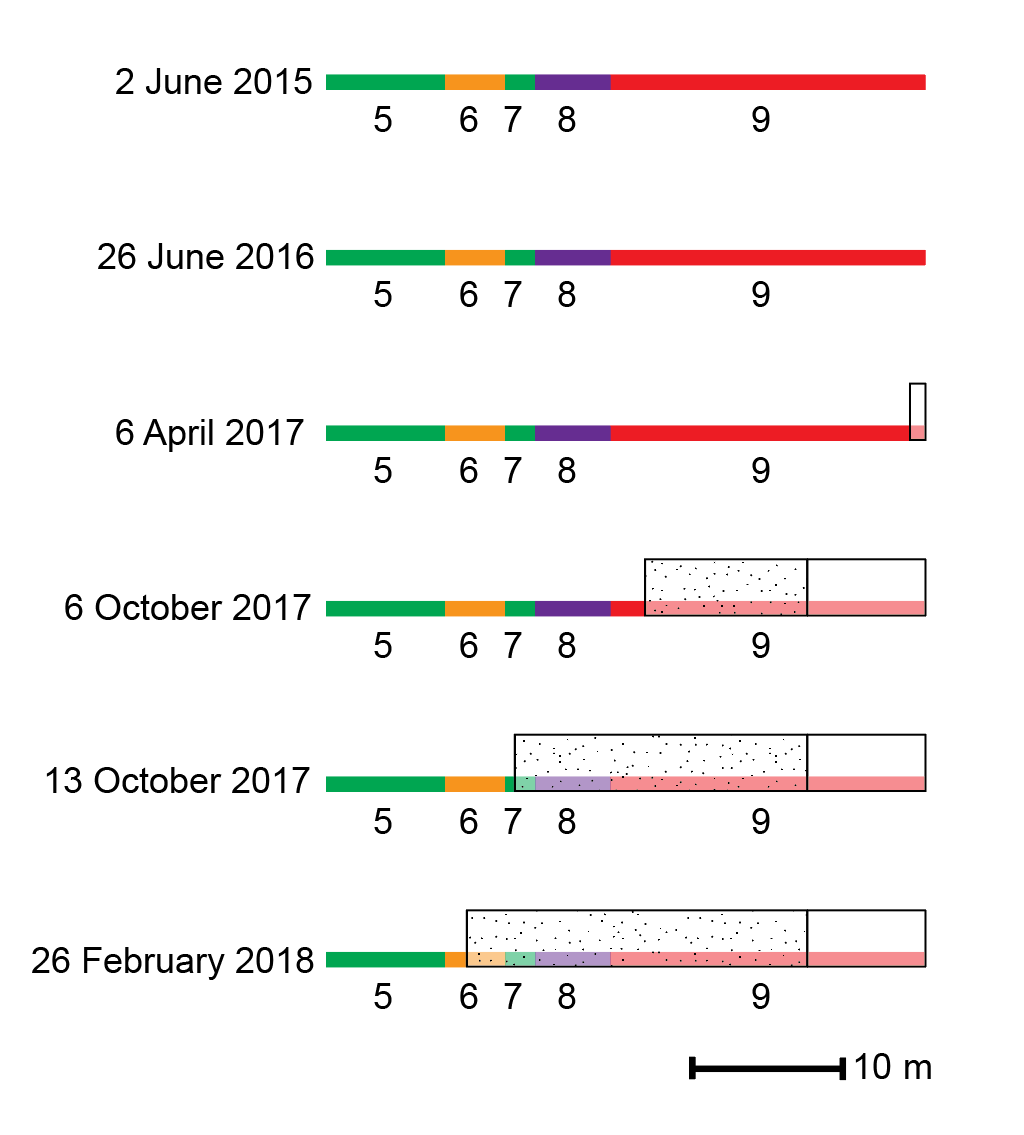


Supplemental Figure 1: Depiction of erosion and overwash on the model cells of Transect A. White boxes represent area lost to erosion, dotted boxes represent area covered by overwash.

References

1. Connor, R. F., Chmura, G. L. & Beecher, C. B. carbon accumulation in Bay of Fundy saltmarshes: Implications for restoration of reclaimed marshes. *Global Biogeochem. Cycles* **15,** 943–954 (2001).
2. Martina, J. P., Currie, W. S., Goldberg, D. E. & Elgersma, K. J. Nitrogen loading leads to increased carbon accretion in both invaded and uninvaded coastal wetlands. *Ecosphere* **7,** (2016).
3. Chrzastowski, M. J., Thompson, T. A. & Brian Trask, C. Coastal geomorphology and littoral cell divisions along the Illinois-Indiana coast of Lake Michigan. *J. Great Lakes Res.* **20,** 27–43 (1994).
4. Terpstra, P. D. & Chrzastowski, M. J. Geometric trends in the evolution of a small log-spiral embayment on the Illinois shore of Lake Michigan. *Source J. Coast. Res. J. Coast. Res.* **8,** 603–617 (1992).
5. Larson, G. & Shaetzl, R. Origin and evolution of the Great Lakes. *J. Great Lakes Res.* **27,** 518–546 (2001).
6. Herdendorf, C. E. Morphometric factors in the formation of Great Lakes coastal wetlands. in *Aquatic Ecosystem Health and Management* **7,** 179–197 (2004).
7. Keough, J. R., Thompson, T. A., Guntenspergen, G. R. & Wilcox, D. A. Hydrogeomorphic factors and ecosystems responses in coastal wetlands of the Great Lakes. *Wetlands* **19,** 821–834 (1999).
8. Hester, N. C. & Fraser, G. S. Sedimentology of beach ridge complex and its significance in land-use planning. *Environ. Geol. Notes* **63,** 1–24 (1973).
9. Larsen, C. E. A stratigraphic study of the beach features on the southwestern shore of Lake Michigan: New evidence of Holocene lake level fluctuations. *Environ. Geol. Notes 112* **112,** 31 (1985).
